# Intermediate Monocytes and High Levels of Chemokine CCL3 Are Associated With Increased Risk of Atrial Fibrillation in the General Population

**DOI:** 10.1161/CIRCEP.124.013621

**Published:** 2025-04-17

**Authors:** Kari Anne Sveen, J. Gustav Smith, Isabel Goncalves, Andreas Edsfeldt, Daniel Engelbertsen, Linda S. Johnson, Olle Melander, Gunnar Engström, Jan Nilsson, Harry Björkbacka, Eva Bengtsson

**Affiliations:** Department of Endocrinology, Morbid Obesity and Preventive Medicine, Oslo University Hospital, Oslo, Norway (K.A.S.).; Department of Cardiology, Clinical Sciences, Lund University and Skåne University Hospital, Lund, Sweden (J.G.S.).; The Wallenberg Laboratory/Department of Molecular and Clinical Medicine, Institute of Medicine, Gothenburg University and the Department of Cardiology, Sahlgrenska University Hospital, Gothenburg, Sweden (J.G.S.).; Wallenberg Center for Molecular Medicine, Lund University, Lund, Sweden (J.G.S., A.E.).; Lund University Diabetes Center, Lund University, Lund, Sweden (J.G.S., I.G., A.E., O.M., J.N., H.B., E.B.).; Department of Clinical Sciences, Malmö, Lund University, Sweden (I.G., A.E., D.E., L.S.J., O.M., G.E., J.N., H.B., E.B.).; Department of Cardiology, Skåne University Hospital, Sweden (I.G., A.E.).; Department of Internal Medicine, Skåne University Hospital, Sweden (O.M.).; Department of Biomedical Science, Faculty of Health and Society, Malmö University, Malmö, Sweden (E.B.).; Biofilms – Research Center for Biointerfaces, Malmö University, Malmö, Sweden (E.B.).

**Keywords:** atrial fibrillation, chemokines, monocytes

Atrial fibrillation (AF) is increasing in prevalence and linked to higher morbidity and mortality rates. Emerging evidence implicates inflammation as a key driver of AF pathogenesis.^1^ Our study aimed to investigate associations between circulating monocyte subsets, monocyte-attracting chemokines and incidence of AF in a large community-based cohort.

The Malmö Diet and Cancer Cardiovascular Cohort is a prospective cohort study conducted between 1991 and 1994.^2^ Incident AF cases were recorded until December 31, 2014, using national registers.^3^ The study was approved by the Swedish ethical review authority, and all participants gave informed consent. Blood monocyte subsets, analyzed by flow cytometry in 666 randomly selected individuals from mononuclear leukocytes frozen at baseline,^2^ were defined as classical (CD14^++^CD16^−^), intermediate (CD14^++^CD16^+^), and nonclassical (CD14^+^CD16^++^). Percentage monocyte subsets were calculated as a percentage of total monocytes (classical, intermediate, and nonclassical). Chemokines (MCP1 [monocyte chemoattractant protein-1], CX3CL1 [CX3C motif chemokine ligand 1], CCL4 [CC motif chemokine ligand], CCL3) and IL-6 (interleukin-6) were measured in plasma using proximity extension analysis (Proseek Multiplex Cardiovascular Panel I; Olink Proteomics, Sweden; n=4704). Monocyte and chemokine analyses overlapped in 528 subjects (Figure [A]). Plasma TNFα (tumor necrosis factor-α) was analyzed in the overlapping subjects using multiplex immunoassay (Millipore). Metabolic syndrome subjects were classified according to the NCEP ATP III definition. Associations with incident AF over time were analyzed by log-rank test and Kaplan-Meier curves. Hazard ratios (95% CIs) were calculated using Cox proportional hazards regression adjusted according to model 1 (age and sex), model 2 (age, sex, clinical risk factors, and prevalent cardiovascular disease; Figure [C]), model 2+cytokines (TNFα, IL-6), or model 2+chemokines (MCP1, CCL3). Proportional hazards assumptions were assessed using Cox regression with time-dependent covariate. *International Classification of Diseases* codes were previously reported.^4^ Correlations were assessed using Spearman rank correlations. The data are available upon reasonable request.

**Figure.**
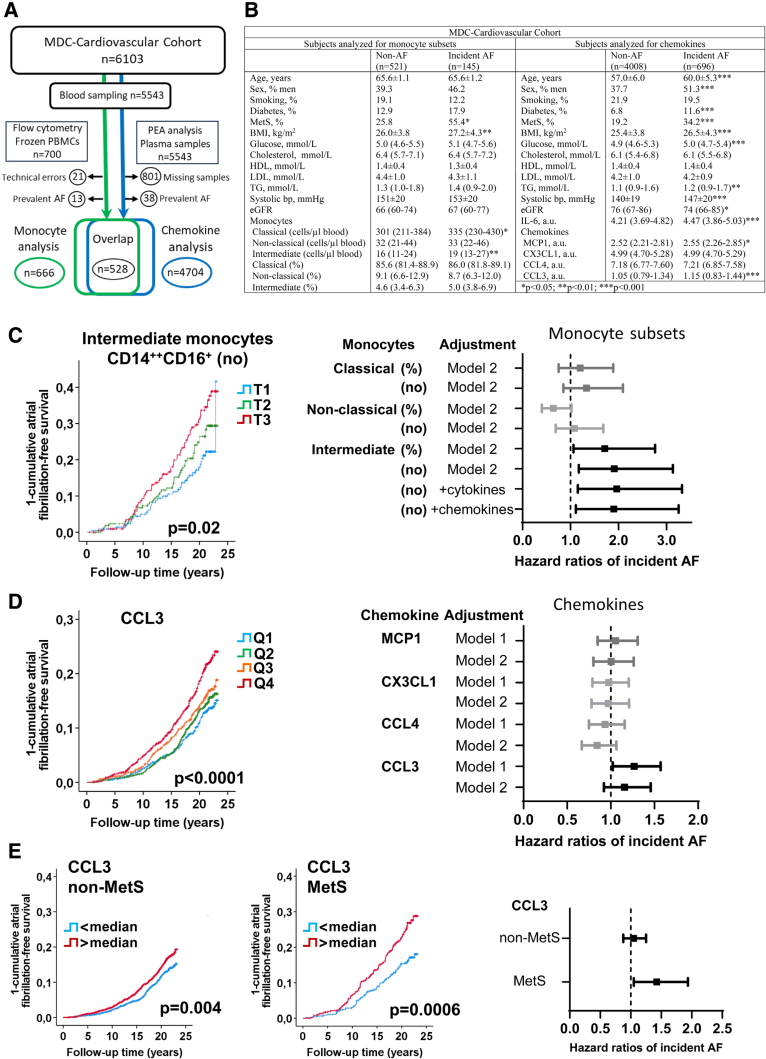
**Intermediate CD14^++^CD16^+^ monocytes and chemokine CCL3 (CC motif chemokine ligand 3) are associated with increased risk of atrial fibrillation (AF). A**, Overview of MDC-cardiovascular cohort. **B**, Baseline characteristics of subjects with and without incident AF. Incident AF cases were recorded during median 18.6 years (subjects analyzed for monocyte subsets) and 21.4 years (subjects analyzed for chemokines) follow-up. **C**, Intermediate monocytes are associated with increased risk of developing AF depicted in Kaplan-Meier curves, tertile 1 to tertile 3 (T1–T3), lowest to highest, log-rank test. HR of incident AF with 95% CI of highest vs lowest tertile of intermediate monocytes (numbers or percentages) adjusted according to model 2 (age, sex, systolic blood pressure, current smoking, body mass index [BMI], triglycerides, high-density lipoprotein [HDL]-cholesterol, glucose, glomerular filtration rate, prevalent coronary event, stroke, and heart failure, and presence of carotid plaque), model 2+cytokines (TNFα and IL-6), or model 2+chemokines (CCL3 and MCP1 [monocyte chemoattractant protein-1]). **D**, CCL3 is associated with increased risk of developing AF, depicted in Kaplan-Meier curves, quartile 1 to quartile 4 (Q1–Q4), lowest to highest, log-rank test. HR of incident AF with 95% CI of highest vs lowest quartile adjusted according to model 1 (age and sex) or model 2 (as above). **E**, CCL3 is associated with increased risk of developing AF in subjects with metabolic syndrome (MetS) depicted in Kaplan-Meier curves, below (<) and above (>) median in subjects with (n=937) or without (n=3661) metabolic syndrome. Hazard ratio of incident AF with 95% CI of above vs below median levels of CCL3 adjusted according to model 1 (age and sex).

Incident AF cases demonstrated elevated levels of intermediate and classical monocytes compared with non-AF subjects (Figure [B]). However, only intermediate monocytes were associated with incident AF after adjusting for risk factors and prevalent cardiovascular disease (Figure [C], model 2). The association remained in a sensitivity analysis excluding subjects with prevalent coronary events, strokes, and heart failures (hazard ratio, 1.82 [1.12–2.97]), number of monocytes, tertile 3 versus tertile 1, *P* =0.017). To take vascular disease over time into account, incident coronary events and heart failures that occurred prior to incident AF were censored. Importantly, the association between incident AF and numbers of intermediate monocytes remained and was even stronger (hazard ratio 2.21 [1.30–3.75], tertile 3 versus tertile 1, *P* =0.003).

Next, we analyzed if monocyte-attracting chemokines were associated with incident AF. Classical monocytes express chemokine receptor CCR2 (CC motif chemokine receptor) and are recruited to sites of inflammation by MCP1. Nonclassical monocytes express high levels of CX3CR1 (CX3C motif chemokine receptor 1) and patrol the vasculature, attracted by CX3CL1. Intermediate monocytes release proinflammatory cytokines and express CCR2, CX3CR1 and high levels of CCR5, which binds to a variety of chemokines, including CCL3 and CCL4. In the present study, MCP1 and CCL3 were elevated in incident AF cases (Figure [B]). After age and sex adjustment, only CCL3 remained associated with incident AF; however, the association was lost after the addition of clinical risk factors (Figure D). Notably, CCL3 correlated with factors of the metabolic syndrome: waist (*r* =0.27, *P* <0.001), glucose (*r* =0.19, *P* <0.001), triglycerides (*r* =0.25, *P* <0.001), systolic blood pressure (*r* =0.17, *P* <0.001), and inversely with high-density lipoprotein (*r* =−0.24, *P* <0.001). Subjects with metabolic syndrome had higher levels of CCL3 than nonmetabolic syndrome subjects (1.23 [0.99–1.55] versus 1.01 [0.75–1.31] arbitrary units; *P* <0.001). Interestingly, the association of CCL3 with AF was stronger in individuals with metabolic syndrome (Figure [E]).

Finally, we assessed correlations of intermediate monocytes with inflammatory cytokines and chemokines. In line with the role of intermediate monocytes in inflammatory cytokine secretion and migration into tissues via CCL3, intermediate monocytes correlated with TNFα (*r* =0.12, *P* =0.007) and CCL3 (*r* =0.09, *P* =0.04). Furthermore, IL-6 levels were higher in incident AF cases (Figure [B]). Importantly, the association of intermediate monocytes with incident AF remained after the addition of plasma cytokines or chemokines to the regression model (Figure [C]).

Our findings highlight the independent association between intermediate monocytes, characterized by their proinflammatory profile, and incident AF. The study supports prior murine studies of a role of monocytes/macrophages contributing to AF.^1^ While classical monocytes were also elevated in incident AF cases, their lack of association in multivariable models suggests a less important role. It is possible that locally enhanced secretion of proinflammatory cytokines by intermediate monocytes may explain the specific association of this monocyte subtype with AF. Our results emphasize the role of chemokine CCL3 in AF, particularly in individuals with metabolic syndrome. Obesity increases the risk of AF and is associated with an increased amount of epicardial fat, producing proinflammatory mediators.^5^ CCL3, expressed by adipocytes, may thus contribute to inflammatory cell recruitment into the myocardium.

This study supports a role of intermediate monocytes and CCL3 in the inflammatory pathway leading to AF. Future research should explore whether reducing inflammation through lifestyle or pharmacological interventions targeting monocytes and chemokines can mitigate AF risk.

## Article Information

### Acknowledgments

The authors are grateful to PhD Katarina Berg, Linda Andersson, and Irena Ljungcrantz for excellent work in measuring monocyte subsets.

### Sources of Funding

This study was supported by Swedish Heart Lung Foundation, the Swedish Research Council, Swedish Governmental Funding of Clinical Research, the Crafoord foundation, Albert Påhlsson foundation, Knut and Alice Wallenberg foundation to the Wallenberg Center for Molecular Medicine in Lund, Skåne University Hospital Funds, Swedish Foundation for Strategic Research to Lund University Diabetes Center, and the Swedish Society for Medical Research.

### Disclosures

Dr Johnson received consulting fees from MEDICALgortihmics and Pfizer. The other authors report no conflicts.
